# Lutetium-177-Labeled Prostate-Specific Membrane Antigen-617 for Molecular Imaging and Targeted Radioligand Therapy of Prostate Cancer

**DOI:** 10.34172/apb.2023.079

**Published:** 2023-04-29

**Authors:** Rien Ritawidya, Hendris Wongso, Nurmaya Effendi, Anung Pujiyanto, Wening Lestari, Herlan Setiawan, Titis Sekar Humani

**Affiliations:** ^1^Research Center for Radioisotope, Radiopharmaceutical, and Biodosimetry Technology, National Research and Innovation Agency (BRIN), Kawasan Puspiptek, Setu, Tangerang Selatan, 15314 Indonesia.; ^2^Research Collaboration Center for Theranostic Radiopharmaceuticals, National Research and Innovation Agency, Jl. Raya Bandung-Sumedang KM 21, Sumedang, 45363, Indonesia.; ^3^Faculty of Pharmacy, University of Muslim Indonesia, Kampus II UMI, Jl. Urip Sumoharjo No.225, Panaikang, Panakkukang, Kota, Makassar, Sulawesi Selatan 90231.

**Keywords:** Prostate cancer, Metastatic-castration resistant prostate cancer, Lutetium-177, PSMA-617, Radioligand

## Abstract

Prostate-specific membrane antigen (PSMA) represents a promising target for PSMA-overexpressing diseases, especially prostate cancer-a common type of cancer among men worldwide. In response to the challenges in tackling prostate cancers, several promising PSMA inhibitors from a variety of molecular scaffolds (e.g., phosphorous-, thiol-, and urea-based molecules) have been developed. In addition, PSMA inhibitors bearing macrocyclic chelators have attracted interest due to their favorable pharmacokinetic properties. Recently, conjugating a small PSMA molecule inhibitor-bearing 1,4,7,10-tetraazacyclododecane-1,4,7,10-tetraacetic acid (DOTA) chelator, as exemplified by [^177^Lu]Lu-PSMA-617 could serve as a molecular imaging probe and targeted radioligand therapy (TRT) of metastatic castration resistant prostate cancer (mCRPC). Hence, studies related to mCRPC have drawn global attention. In this review, the recent development of PSMA ligand-617-labeled with ^177^Lu for the management of mCRPC is presented. Its molecular mechanism of action, safety, efficacy, and future direction are also described.

## Introduction

 Prostate cancer is the second most frequent type of cancer among men in the world.^1^ The incidence rate of this type of malignancy varies worldwide and it is considered the leading cause of mortality in men. According to the Global Cancer Observatory: Cancer Today (GLOBOCAN), in 2020, there were estimated 1 414 259 (7.3%) incidences occurred across countries, with a number of mortalities estimated 375 304 (3.8%).^2^ This situation reflects how prostate cancer has become a major health problem on a global scale.

 Treatment options available for prostate cancer in the early stages of the disease progression mainly rely on surgery, external beam radiation therapy, and brachytherapy,^3^ while other treatments such as hormone therapy, chemotherapy, and radiation therapy administered alone or in combination, are usually considered for the treatment of malignant metastases or as additional therapies in the early stages of prostate cancer.^3,4^ Androgen-deprivation therapy is emerging as the first-line treatment for advanced prostate cancer.^5-7^ However, in most cases, there can be clinical and biochemical progression of this cancer and this condition is termed metastatic castration-resistant prostate cancer (mCRPC).^8,9^ The most common treatment options at this stage include docetaxel, sipuleucel-T, abiraterone and radium-223 (Xofigo^TM^).^9,10^ However, this approach is known to lead to suboptimal results.^11^ Recently, the poly(ADP-ribose) polymerase inhibitors, such as olaparib and rucaparib have been evaluated in phase 2 clinical trials as novel therapy for mCRPC with tumors lacking homologous recombinant repair.^12^ Olaparib and rucaparib have been approved and shown to be effective in mCRPC patients with BCA1/2 abnormalities.^12^ Despite the progress and emergence of various therapeutic methods, an effective treatment approach with minimal side effects for mCRPC is still needed.

 The serum prostate-specific antigen (PSA) screening test and the digital rectal examination are widely used methods to detect the pathology of prostate cancer.^11^ PSA level cut-off of 4.0 ng/mL has been used to decide the need for prostate biopsies.^13^ While transrectal ultrasound (TRUS)-guided multiple systematic transrectal biopsies are typically performed for the diagnosis purposes by obtaining the tissue sample from the gland for histopathological or cytological examination.^4^ Several imaging techniques, such as magnetic resonance imaging (MRI) and positron emission tomography (PET) play a pivotal role in the management of prostate cancer, especially for early detection and localization, (re-)staging, whole-gland and focal therapy, active surveillance, and detection of recurrence.^14,15^ In addition to PET, the single-photon emission computed tomography (SPECT) modality enables nuclear diagnostic imaging in prostate cancer. Consequently, the advancement of PET and SPECT modalities led to the necessity of efficient imaging agents or radiopharmaceuticals probes that would enable the detection of prostate cancer.

 Prostate-specific membrane antigen (PSMA) is a type II transmembrane glycoprotein (~100 kDa) highly expressed in prostate cancer^16^ and upregulated in poorly differentiated, metastatic, and hormone-refractory carcinoma, castration-resistant prostate cancer.^17^ In addition, organ-minimally expressing PSMA can be found in various organs, including the brain, kidney, salivary gland, and intestine.^18^ PSMA is known to possess neurocarboxypeptidase activities that degrade alpha-linked glutamates from *N*-acetylaspartylglutamate^19^ in addition to its prominent role as folate hydrolase I.^20^ PSMA also plays an important role in angiogenesis.^21^ Accordingly, PSMA has recently gained growing interest as a promising target for diagnostic imaging and therapy of prostate cancer.^1,22^

**Figure 1:**
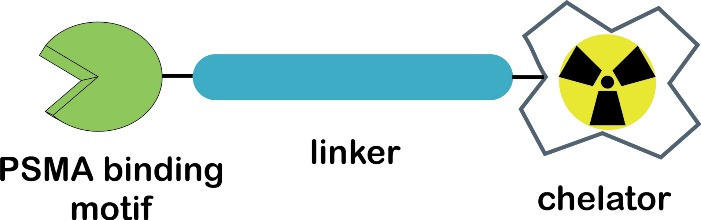
General design of PSMA-targeted radioligand.^27,28^

 Targeted radioligand therapy (TRT) is a selective or specific administration of a high dose of radiotoxicity to cancer cells without destroying the surrounding healthy cells.^23,24^ It typically employs targeting vectors such as proteins, peptides, carbohydrates, vitamins, antibodies, and aptamers.^25^ Metal-based small-molecule PSMA radioligands have shown a growing interest in TRT prostate cancer.^26^ A common strategy to develop PSMA-specific based radiometal ligands is shown in Figure 1.^27,28^

 A macrocyclic chelator 1,4,7,10-tetraazacyclododecane-1,4,7,10-tetraacetic acid (DOTA) is widely used in the field of radiopharmaceuticals, particularly for the complexation of trivalent (3 + ) ions such as the diagnostic PET radionuclide ^68^Ga and therapeutic radionuclides (^177^Lu and ^90^Y).^26,28,29^ The presence of linkers can connect two different moieties: a chelating agent and a pharmacophore.^30^ Complexation of DOTA and a trivalent radiometal resulted in a thermodynamically and kinetically stable binding.^28^ Furthermore, this approach allows that the theranostic concept in nuclear medicine, which defines ideal radiopharmaceuticals should be able to assemble the application for both diagnostic and therapeutic purposes when radiolabeled with a diagnostic and a therapeutic radionuclide, respectively.^26,31^

## PSMA

**Figure 2:**
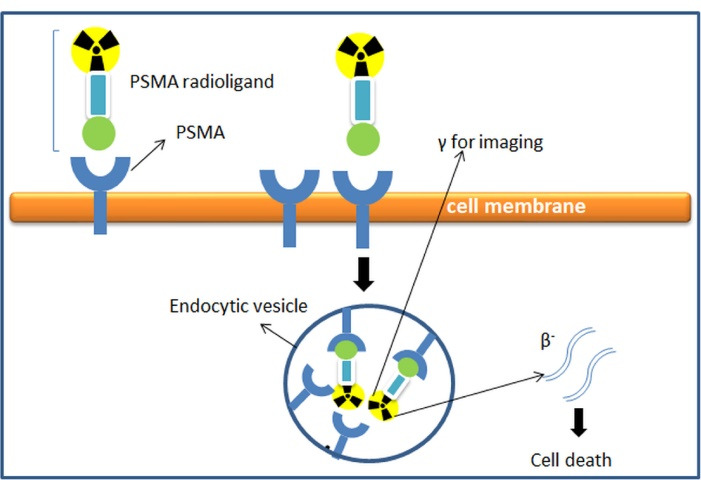
Mechanism of receptor-mediated endocytosis upon radioligand-based PSMA binding to PSMA.^27^

 PSMA has emerged as a promising protein target for prostate cancer for both diagnosis and therapeutic purposes (e.g., radionuclide-based therapy or other therapeutic strategies including immunotoxins, immune cells retargeting, prodrug activation, PSMA vaccines, plasmid DNA, and adenoviral immunizations.^30-32^ This mechanism leads to the internalization of radionuclides into the cancer cells and eventually causes cell death^33^ as shown in Figure 2. The unique characteristics of PSMA make it an excellent marker for prostate cancer, mainly due to several characteristics including: 1) expressed in the prostate, 2) upregulated in all stages of the disease, 3) overexpressed in disease progression or in metastases, 4) intact on the cell surface as membrane glycoproteins, present and not released into the circulation, 5) internalized after ligand binding (receptor-mediated endocytosis), 6) associated with enzymatic activity.^3,18,23^

 PSMA shares sequence similarities to a certain extent (~54%) with transferrin receptors,^18,34^ and therefore, like transferrin, PSMA undergoes receptor and ligand functions.^18^ Immunofluorescence analysis or immunoelectron microscopy shows that after ligand binding, the PSMA-antibody complex is internalized through clathrin-coated pits and enters the lysosomes.^34^

## Radiolabeled PSMA

**Figure 3:**
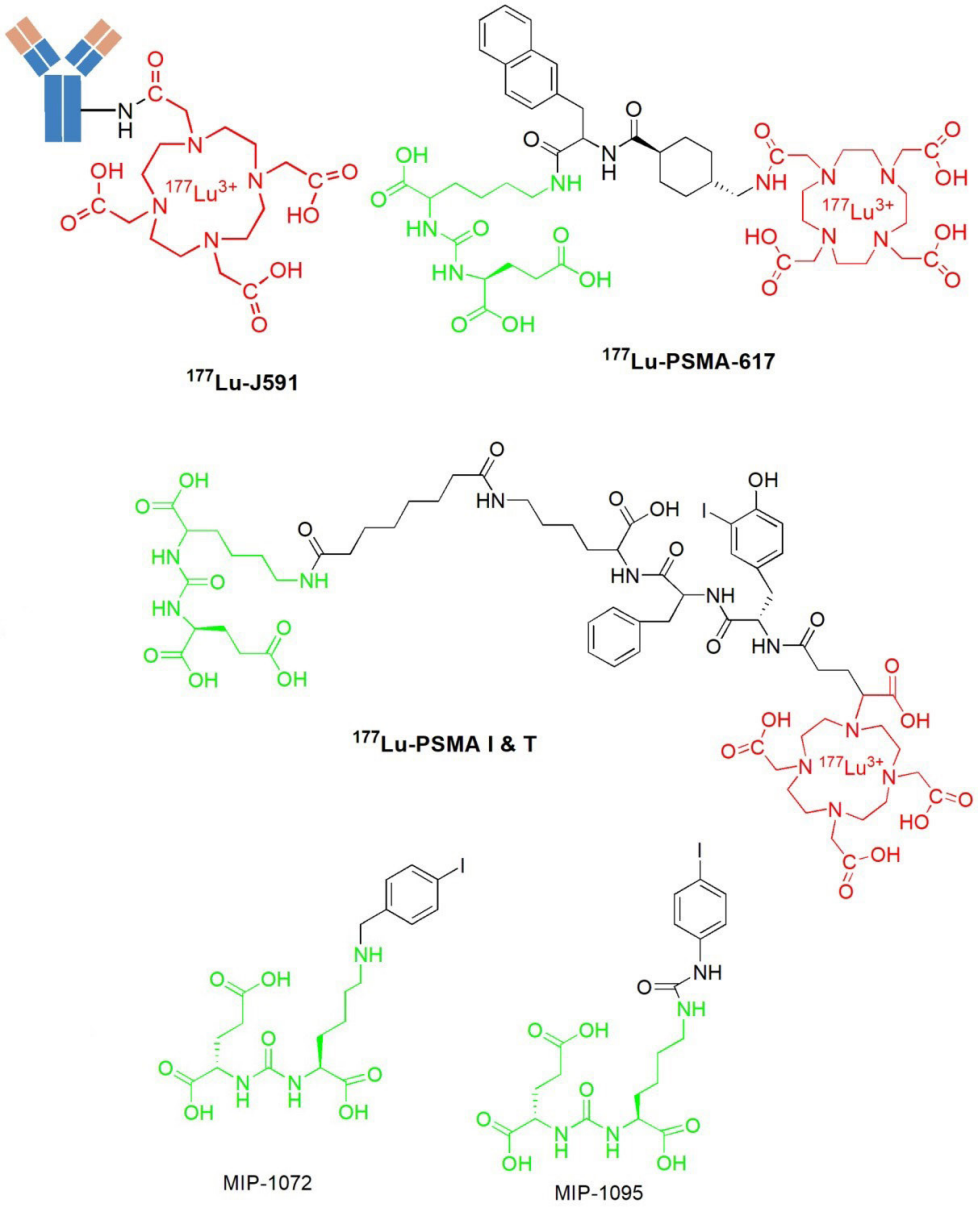
Some radiolabeled PSMA ligands

 A radiolabeled monoclonal antibody ProstaScint^TM^ (Capromab Pendetide) is a murine IgG1 7E11-C5.3 which is linked to a linker-chelator glycyl-tyrosyl-(N’- diethylenetriaminepentaacetic acid)-lysine hydrochloride^35^ and it was developed to accurately diagnose, stage, and detect the new and recurrent prostate cancer.^36^ ProstaScint^TM^ targets PSMA by binding to the intracellular domain (amino-terminus) of PSMA^35^ and areas of tumor necrosis.^18^ Accordingly, this radiotracer found limited use in nuclear medicine to diagnose prostate cancer.^26^ The development of monoclonal antibodies J591 that bind to the extracellular domain of PSMA has been reported in the literature. The J591 monoclonal antibody demonstrated high and specific binding against cell-adherent PSMA.^37^ In addition, J591 was the first PSMA-based humanized monoclonal antibody used in the clinical application.^38,39^ Several SPECT and PET tracer-based J591,^40-42^ as well as radioimmunotherapeutic agents have been developed.^43^ Some of the PSMA-specific radioligands studied so far are shown in Figure 3.

 However, the nature of the monoclonal antibody, including slow clearance and low uptake, underlines the need for imaging to be performed several days after its administration to patients.^39^ Therefore, the waiting time between post-administration and the imaging time seems to hinder the potential application of this PSMA-targeted J591 monoclonal antibody.^39,44^

**Figure 4:**
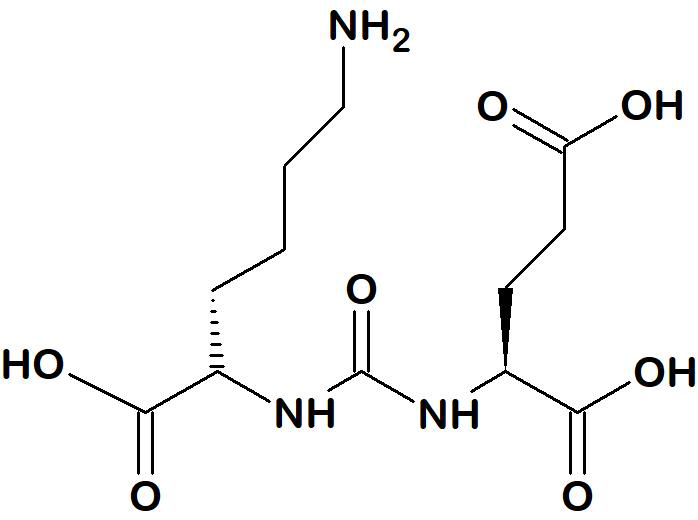
Lys-urea-Glu (K-u-E) binding motif

 Continued efforts to discover several specific-PSMA inhibitors with a higher affinity and specificity for PSMA led to various small molecule inhibitors. Small molecule PSMA inhibitors are typically zinc-binding compounds incorporated into glutamate or glutamate isostere and are divided into three classes: 1) phosphonate, phosphate, and phosphoramide compounds; 2) thiols; and 3) ureas (Figure 4).^45-47^ The phosphorus-based ligands seem to be the gold standard that provide binding to binuclear zinc ions positioned in the active PSMA domain. However, the development of these ligands is limited by their high polarity properties. PSMA ligands bearing thiol functionality, on the other hand, could undergo disulfide bond formation, resulting in low metabolic stability. Thus, some urea-based PSMA ligands have been developed. These molecules display favorable binding affinity and stability with very efficient internalization into the cells.^46-48^

 The first urea-based compound to target PSMA in the brain was designed by Kozikowski et al.^49^ To date, urea-based PSMA radiopharmaceuticals are the most sophisticated class which is commonly consisting of three parts, a binding motif (glutamate-urea-lysine [Glu-urea-Lys]), a linker, and a radiolabeled moiety (usually a chelator or prosthetic groups) depending on the radionuclide.^23^

 Liu et al evaluated the dependence of linker length on inhibitory potency, mode of inhibition, and in vitro imaging of three different fluorescent inhibitors.^50^ They found that choosing the right linker, along with its length, are such crucial considerations in the development of PSMA detection probes and therapy tracers that specifically target PSMA-overexpressing cells.^50^

 The discovery of new developed radioiodinated, ^123^I-MIP-1072 and ^123^I-MIP-1095 (Figure 3) PSMA ligands based on urea scaffold have been reported in the literature.^25,51-53^ Despite the encouraging earlier clinical results, it appears that further attempts to optimize the efficacy and reduce the side effects of these radioiodinated ligands are warranted.^30^ As a result, the development of ^123^I-MIP-1072 and ^123^I-MIP-1095 has initiated the development of other PSMA-based urea binding motif radiopharmaceuticals eligible for prostate cancer.^23^

 The radiometal-based PSMA binding motif [Glu-Urea-Lys] has shown a growing interest in the endoradiotherapy of prostate cancer.^26^ Due to its favorable coordination chemistry properties, the DOTA chelator can be used to conjugate several radiometals, including ^177^Lu and ^68^Ga, whereas the linker can connect two different moieties: chelator and pharmacophore.^30^ In 2014, a research group in Munich reported the development of the metabolically resistant 1,4,7,10-tetraazacyclododecane,1-(glutaric acid)-4,7,10-triacetic acid (DOTAGA) chelator moiety based on their previously advanced affinity PSMA ligand [^68^Ga]Ga-DOTAFfK(Sub-KuE)).^54^ In 2015, a research group in Heidelberg developed a DOTA-containing PSMA inhibitor, PSMA-617.^30^ This PSMA-617 contains three molecule entities, which are the pharmacophore (binding motif), glutamate-urea-lysine; the chelating agent DOTA, and a linker connecting these two moieties.^30^ The presence of a linker in peptide-based radiopharmaceuticals can improve metabolic stability and modulate the biodistribution.^55^ In addition, the linker plays an important role in bridging between a chelator and a pharmacophore; thereby maintaining peptide affinity for the receptor and avoiding the steric hindrance.^56^ The linker can trigger multiple effects by modulating the size, shape, solubility, stability, and molecular weight of the chemical structure, which positively aids the overall radiopharmaceutical behaviours.^57^ Benesová et al investigated the influence of chemically modified linkers on PSMA targeting and the pharmacokinetic profile, including PSMA inhibitory activity, cellular internalization, and biodistribution properties of a series of DOTA-PSMA small molecules.^58^ The study approach led to a more accurate and rational structure-activity relationships design of a new specific PSMA-based glutamate-urea motif, resulting in a promising DOTA-PSMA conjugate that can potentially be radiolabeled for theranostic application of prostate cancer.^58^

 Numerous attempts have been made by the scientific community to develop various PSMA radionuclides based on PSMA ligands. Of several radiolabeled ligands reported in the literature, the radiopharmaceutical ^177^Lu-PSMA-617 has been one of the most extensively studied PSMA radioligands for both prostate cancer imaging and therapy. Phase III clinical trials of radioligand VISION (^177^Lu-PSMA-617, NCT03511664) is currently being conducted.^59^ Accordingly, the presence of extensive knowledge, experience, and information related to this radiopharmaceutical leads us to develop an “in-house” PSMA-617-based-radioligand devoted to the management of metastatic prostate cancer in Indonesia. In this review, the recent development of PSMA ligand-617-labeled with ^177^Lu for the management of mCRPC is presented. Its molecular mechanism of action, safety, efficacy, and future direction are also described.

**Figure 5:**
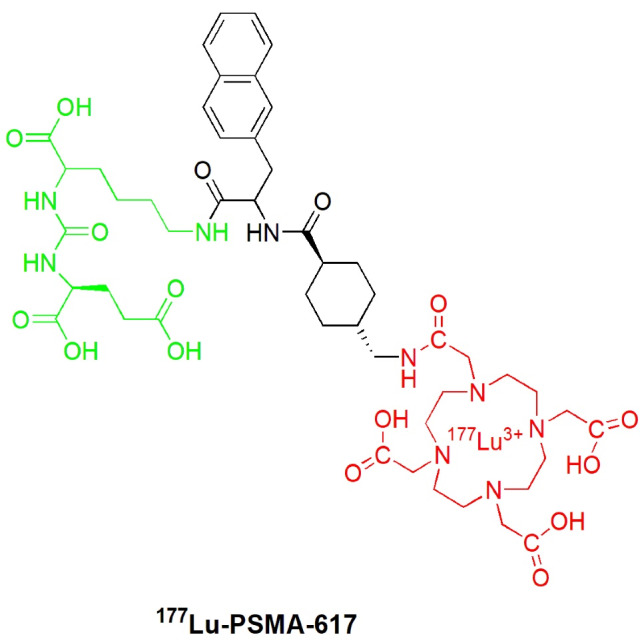
Structure of ^177^Lu-PSMA-617 radioligand.

 Recently, ^177^Lu-PSMA-617 (Figure 5) was a novel promising radiopharmaceutical for nuclear imaging and TRT that is reported to be safe and can prolong overall survival in mCRPC patients.^60-64^ The development of this urea-based small PSMA inhibitor labeled with a beta particle-emitting radionuclide (Lu-177) was initially performed by a research group from the German Cancer Research Center (Deutsche Krebforschungszentrum, DKFZ) and its collaborating partner, the University Hospital of Heidelberg Germany in 2015.^30^

 The PSMA-617 ligand was synthesized by the solid phase peptide method as described in the previous literature.^65^ Small peptides represent several advantages over monoclonal antibodies, including high penetration, better pharmacokinetics, high affinity and specificity for the target site.^66,67^ These features often resulted in a higher target-to-non-target ratio, which is important for both imaging and the successful therapeutic application of absorbed dose.^68^

 This custom-designed DOTA containing the small PSMA inhibitor PSMA-617 was reported to be successfully radiolabeled with ^177^Lu in a small amount (0.5 mg, 0.5 nmol) in sodium acetate buffer, pH 5 with an excellent radiochemical yield ( > 99%).^30^ The preparation of ^177^Lu-PSMA-617 is also described in the literature.^69^ The ^177^Lu-PSMA-617 prepared “in-house” by our group resulted in a comparable radiochemical yield of more than 99% (data not reported), which is consistent with that reported in the literature.

## ^177^Lutetium radionuclide

 Therapeutic radionuclides fall into three classification groups, namely beta particles (β^-^), alpha emitter (α), and Auger electron.^70^ Of these therapeutic radionuclides for targeted therapy, the beta particles emitter ^177^Lu has gained remarkable applications in recent years.^70^

^177^Lu can be routinely produced in high activity levels with a high specific activity in a nuclear reactor available worldwide.^70^ Although ^177^Lu can be crafted in a particle-accelerating machine or cyclotron,^71^ nuclear reactor production via neutron activation is preferred. Two methods for ^177^Lu production via a nuclear reactor are available, including a direct method and an indirect method.^72^ The direct method production or carrier-added approach employs enriched ^176^Lu as the irradiation target. While the latter one uses an enriched ytterbium (^176^Yb) target for irradiation.^72,73^ High specific activity of ^177^Lu is of great importance for the application of TRT, especially for the production of various therapeutic radiopharmaceuticals based on peptides and antibodies.^72^ The generator-based production of ^177^Lu from its long-live isomer ^177m^Lu was reported.^70,74^ In addition to the generator radionuclide approach, the separation method of ^177^Lu from chemically and physically similar ^177m^Lu based on the nuclear after-effect of nuclear decay was described.^75^

^177^Lu emits β^-^ particles for therapeutic disease purposes and its γ emission is useful for SPECT imaging. The cross-fire effect of ^177^Lu has pointed this radionuclide as a suitable radionuclide for targeted therapy of various malignant disorders.^63,76^ The physical and chemical properties of ^177^Lu (t_1/2_ = 6.73 days, E_βmax_ = 497 keV, E_γ_ = 113 keV (6.4%) and 208 keV (11%)) makes it a favorable radionuclide for the development of therapeutic radiopharmaceuticals. Its β^-^ particle energy (0.5 MeV maximum energy β-emission) allows the delivery of radiotoxicity specifically towards the tumors rather than the healthy tissue.^77^ The range of ^177^Lu penetration towards the tissue is appropriate for small tumors ( < 2 mm) and metastases compared to the longer penetration of yttrium-90 (12 mm), and may result in minimal kidney radiation exposure.^77,78^ Its cross-fire effect has become the important mechanism of the therapeutic outcome of radioligand therapy by destroying the surrounding cells of tracer-accumulating cells.^79^ Additionally, its lower gamma emission is sufficient for SPECT imaging allowing *in vivo* biodistribution imaging and pharmacokinetic studies as well as dosimetry measurements.^72^

 Considerable interest in ^177^Lu applications has been growing since an established application ^177^Lu-DOTA-TATE (Lutathera^®^) as a peptide receptor radionuclide therapy (PRRT) radiopharmaceutical for the treatment of somatostatin receptor-positive cancers, such as neuroendocrine tumors.^80^ Lutathera^®^ is the first PRRT radiopharmaceutical and was approved by The European Medicines Agency (EMA) in 2017 and by The Food and Drug Administration (FDA) in 2018.^80^ Preparation of several radiopharmaceuticals based on ^177^Lu has been reported in previous studies.^81–86^ Recently, the potential application of ^177^Lu for therapy of another target receptor, such as the gastrin-releasing peptide receptor (GRPR) has been described.^1,87,88^ GRPR is overexpressed in a variety of cancers such as prostate cancer.^24,89^ Rousseau et al described the development of the GRPR-targeted radiopharmaceutical, ^177^Lu-NeoBOMB1, as a promising radiopharmaceutical for prostate cancer.^87^ The preclinical studies investigating the use of the antagonist GRPR NeoBOMB1 for theranostic usage with ^68^Ga and ^177^Lu were investigated.^90^ The findings showed that ^177^Lu-NeoBOMB1 and ^68^Ga-NeoBOMB1 exhibited significant tumor uptake and favorable pharmacokinetic properties, and therefore can be potentially used as promising radiotracers for imaging and treatment of GRPR-positive cancers.^90^ Kurth et al reported the first human studies of another selective antagonist peptide towards GRPR, RM2-labeled with therapeutic ^177^Lu radionuclide.^88^ ^177^Lu-RM2 has been found effective for treating mCRPC for patients with an insufficient amount of PSMA. Four patients who showed high GRPR expression on ^68^Ga-RM2 PET/CT imaging received ^177^Lu-RM2. The results showed that ^177^Lu-RM2 therapy was considered a safe treatment in terms of radiation safety for both patients and caregivers.^88^ A promising therapeutic application of ^177^Lu-DOTA-trastuzumab for the treatment HER-2-breast cancers was reported.^91^ The planar and SPECT/CT imaging results showed uptake at both the primary as well as the metastatic sites. In addition, the lack of localization of ^177^Lu-DOTA-trastuzumab in negative HER-2 breast cancer patients indicates the specificity of this radiopharmaceutical for treatment of HER-2-positive breast cancer in the future.^91^

## Preclinical and clinical investigations of ^177^Lu

^177^Lu-PSMA-617is characterized by its high radiolytic stability for at least 72 hours, a high inhibitory potency ([Ki] = 2.34 ± 2.94 nM on LNCaP, Ki = 0.37 ± 0.21 nM enzymatically determined), and high internalization into LNCaP cells. In addition, the dynamic small PET imaging demonstrated high tumor-to-background contrast 1 hour p.i. The radiolabeled PSMA-617 also demonstrated rapid renal clearance and favorable pharmacokinetic properties, resulting in very high tumor-to-blood and tumor-to-muscle ratios of 1058 and 529, respectively.^30^

 Clinical studies were conducted to evaluate the potential of this novel radioligand as a radioendotherapeutic agent for prostate cancer. Several multicenters around the world have demonstrated the high response rate as well as the low toxicity achieved after therapy with this ^177^Lu-labeled PSMA-617.^60-62,69,70,92-96^

 In general, the clinical studies investigating the efficacy and safety of ^177^Lu-PSMA-617 are based on retrospective studies in patients with metastatic castration-resistant prostate cancer who have failed three in-line therapies, including chemotherapy, second generation anti-androgen and radium-223.^64^ Table 1 summarizes retrospective clinical trials with ^177^Lu-PSMA-617 in different multicenter.

**Table 1:** Clinical studies of ^177^Lu-PSMA-617 in various multicenter

**Toxicities**	**PSA evaluation**	**Response** **PSA decline (≥50%) (%)**	**Activity range** **per cycle (GBq)**	**n**	**References**
Grade 3–4: anemia (10%); thrombocytopenia (4%); and leukopenia (3%)	2-4 wk after	45/99 (45%)	2-8	99	^61^
Grade 1 dry mouth (87%); grade 3 or 4 thrombocytopenia (13%)	3-4 wk after	17/30 (57%)	4.4-8.7	30	^96^
Grade 3-4 haemotoxicity, leucopenia grade 2	2 months	5/10 (50%)	4.1-6.1	10	^62^
Moderate acute haemotoxicities, grade 1 leucopenia (20%); grade 2 leucopenia (7%)	every 4 wk	13/30 (57%)	3.7-4.0	30	^69^
Mild nausea	every 8 wk	cycle 1 10/24 (41.6%); cycle 2 (59%)	4.1-7.1	24	^60^
Hemoglobin toxicity: grade 2(1) and grade 3(1)	2 wk, 4 wk, and 3 months	Biochemical response: complete 2/31 (6%), partial 20/31 (64.5%)	Mean activity 5.069 ± 1.845	31	^95^
Grade 3 or 4 hematologic toxicity (4) (3.4%)	after one course of PRLT	46/80 (57.5%)	2.0-9.7	119	^94^
Grade 3 leukocytopenia (2) and grade 3 (1) anemia	4 wk after 3^rd^ treatment	31/54 (58%)	7.4	54	^92^
Grade 3 leukocytopenia (2)	every 2 wk	5/14 (36%)	6.0-8.0	14	^93^

 An early report on side effects and the efficacy of this ^177^Lu-PSMA-617 radiotherapeutic agent was published by Ahmadzadehfar et al.^62^ A total number of ten patients involved in this trial received only this radiolabeled agent as a single treatment. The PSA biochemical response was an indication of efficacy and was measured two months after treatment. The tolerability of the therapy was evaluated with regard to the occurrence of post-therapeutic symptoms and toxicities. Notably, seven patients had reduced PSA levels, with 50% of them experiencing a decreased PSA level ( ≥ 50%). No patients showed serious side effects during and after hospitalization. Following this promising initial result, a larger cohort of 24 patients was selected to undergo up to two cycles of ^177^Lu-PSMA-617 radioligand therapy ranging from 4.1-7.1 GBq (mean of 6.0 GBq).^60^ Similar to the previous study, no patient showed side effects immediately after administration of ^177^Lu-PSMA-617. Of 24 patients evaluated 2 months after the first cycle of ^177^Lu-PSMA-617, 19 patients (79.1%) showed decrease PSA level; 13/24 patients (PSA decline by more than 30%) and 41.6% experienced a PSA reduction more than 50%, while 5 patients demonstrated disease progression. Twenty-two of the 24 patients were recruited to undergo a second cycle, and 15 patients (68.2%) experienced a fall in PSA level, with 59% showing more than 50% PSA decline. The most common side effect in the first 2 days after injection was mild nausea (in 3 patients). In the same year, Kratochwil et al conducted retrospective studies in 30 patients.^69^ Each patient received 1-3 cycles of ^177^Lu-PSMA-617. Most patients experienced mild to moderate toxicity.^69^

## PSMA labeled with alpha emitter for targeted alpha therapy (TAT)

 Alpha-labeled-PSMA-617 display a great potential for the treatment of metastatic prostate cancer. Therapeutic alpha-emitting radionuclides such as Ac-225, Tb-149, At-211, Bi-212 (lead-212), Bi-213, Ra-223, and Th-227 have higher energy compared to beta particle-emitting radionuclides and a short penetration path length.^97–99^ Therefore, they present a higher linear energy transfer. A high linear energy transfer of the alpha emitter can lead to the DNA double-strand break when interacting with nuclei. Consequently, compared to the beta emitter, TAT results in a more cytotoxic dose to cancer cells while keeping the dose to the surrounding healthy cells minimal.^59,100^ Kratochwil reported the first human studies of ^225^Ac-PSMA-617 in two patients who showed positive PSMA expression with PET/CT imaging of ^68^Ga-PSMA-11.^101^ After ^225^Ac-PSMA-617 therapy, the patients showed significantly lower PSA levels and complete imaging responses. Despite the remarkable results of ^225^Ac-PSMA-617 therapy, availability, isolation and separation chemistry for ^225^Ac, and stable targeting systems accompanied by a high labeling yield are still considered challenging issues.^102^ Therefore, the application of ^177^Lu-PSMA-617 to treat mCRPC is of great interest. Despite the promising results of ^225^Ac-PSMA-617, only a limited number of clinical studies have been reported. The success of TAT-PSMA therapy also depends on the chelating agents, improved tumor uptake of linkers and targeting vectors, and reduced toxicity and progeny redistribution.^59^ Because PSMA-TAT can potentially lead to xerostomia,^101^ tandem beta (β^-^) emitting ^177^Lu-labeled PSMA may help reduce the occurrence of dose-limiting toxicity, including xerostomia.^103^ In addition, it can lower the ^225^Ac-PSMA-617 and improve the effectiveness of ^177^Lu-PSMA-617.^103^ Recently, Yadav et al studied the efficacy and toxicity of ^225^Ac-PSMA-617.^104^ They reported the promising salvage therapy accompanied by minimal toxicity, indicating the great benefit possibility for mCRPC patients who have failed standard care, including ^177^Lu-PSMA-617.^104^

## Conclusion

^177^Lu-PSMA-617 is a promising radiopharmaceutical for diagnostic imaging and therapeutic of mCRPC. Due to its mild toxicities and suitable in vitro and in vivo properties, this radioligand possesses greater biomedical applications. Therefore, ^177^Lu-PSMA-617 could become the modality of choice for the management of prostate cancer in clinical settings, including oncology and nuclear medicine.

## Acknowledgments

 The authors would like to acknowledge funding from The National Research and Innovation Agency (BRIN) under the Research and Innovation for Advanced Indonesia 2022 scheme (decree number 82/II.7/HK/2022) and The Indonesian Endowment Funds for Education (LPDP). The authors would also like to thank Dr. Tita Puspitasari and Dr. Rohadi Awaludin for their valuable comments on this manuscript.

## Competing Interests

 The authors declare no conflict of interests.

## Ethical Approval

 Not applicable.
